# Quasiparticle self-consistent *GW* study of cuprates: electronic structure, model parameters, and the two-band theory for T_*c*_

**DOI:** 10.1038/srep12050

**Published:** 2015-07-24

**Authors:** Seung Woo Jang, Takao Kotani, Hiori Kino, Kazuhiko Kuroki, Myung Joon Han

**Affiliations:** 1 Department of Physics, Korea Advanced Institute of Science and Technology (KAIST), Daejeon 305-701, Korea; 2 Department of Applied Mathematics and Physics, Tottori University, Tottori 680-8552, Japan; 3 National Institute for Materials Science, Sengen 1-2-1, Tsukuba, Ibaraki 305-0047, Japan; 4 Department of Physics, Osaka University, Machikaneyama-Cho, Toyonaka, Osaka 560-0043, Japan; 5 KAIST Institute for the NanoCentury, Korea Advanced Institute of Science and Technology, Daejeon 305-701, Korea

## Abstract

Despite decades of progress, an understanding of unconventional superconductivity still remains elusive. An important open question is about the material dependence of the superconducting properties. Using the quasiparticle self-consistent *GW* method, we re-examine the electronic structure of copper oxide high-T_*c*_ materials. We show that QSGW captures several important features, distinctive from the conventional LDA results. The energy level splitting between 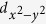 and 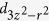 is significantly enlarged and the van Hove singularity point is lowered. The calculated results compare better than LDA with recent experimental results from resonant inelastic xray scattering and angle resolved photoemission experiments. This agreement with the experiments supports the previously suggested two-band theory for the material dependence of the superconducting transition temperature, T_*c*_.

After the seminal work of finding high temperature superconductivity in a ceramic copper oxide material^1^, efforts to understand the cuprate have long been a central part of modern condensed matter physics. Although many intriguing aspects of its electronic behaviors have been unveiled and great progress has been made, an understanding of its superconducting mechanism, the novel interplay between competing phases, and its relationship to other correlated phenomena is still far from clear^2^,^3^,^4^,^5^,^6^,^7^,^8^,^9^,^10^,^11^,^12^. One simple, well-defined but still open question is what determines the superconducting transition temperature (T_*c*_), or its material dependency. For example, the T_*c*_ of the single layer cuprates can be different by a factor of two; ~40 K for La_2_CuO_4_ and ~90 K for HgBa_2_CuO_4_. On the one hand, it may be too early to ask this question, while the superconducting mechanism itself still remains elusive. On the other, however, figuring out the detailed features behind the material dependency can provide the crucial hint for further understanding of superconductivity and other related properties. In fact, many directly or indirectly related theoretical studies have been performed on this issue^13^,^14^,^15^,^16^,^17^,^18^,^19^,^20^,^21^,^22^,^23^,^24^,^25^

In this regard, a notable suggestion has recently been made^26^,^27^,^28^,^29^. According to this theory, the T_*c*_ of the single-layer cuprate can be described with a two-orbital model that considers both the 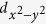 and 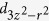 Wannier orbitals, and the energy level offset between the two orbitals, ΔE, plays a key role in determining T_*c*_. Whereas the larger value of this energy separation produces the higher T_*c*_ (*e.g.,* the case of HgBa_2_CuO_4_) due to the better one band feature achieved, the smaller value results in the lower T_*c*_ (*e.g.,* the case of La_2_CuO_4_) in spite of the better nested Fermi surface. The calculated Eliashberg parameter (*λ*) based on the many-body calculation using the fluctuating exchange (FLEX) approximation^30^,^31^ clearly exhibits a linear dependence on ΔE while the other parameters are shown to be less important. Further, this theory can be extended to the bilayer case^29^, which explains the correlation between the Fermi surface shape and T_*c*_^24^.

A possible experimental test to verify this two-band theory is to examine the correlation between T_*c*_ and Δ*E*, the Fermi surface shape, or the partial density of states of the 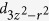 orbital, which can be measured by recent techniques such as resonant inelastic xray scattering (RIXS), and (angle resolved) photoemission spectroscopy (ARPES). However, while the theoretical Δ*E* or the Fermi surface shape was obtained from the LDA and used as the “inputs” for the many-body FLEX calculation in Refs 26, 27, 28, 29, the experimentally determined Δ*E* and the Fermi surface shape should be regarded as the “outputs” or “results” after the consideration of the many-body correlation effects beyond LDA/GGA. In fact, while a RIXS study reports that T_*c*_ is higher for larger Δ*E*^32^, the actual experimental value of Δ*E* is larger than the theoretical evaluation, presumably due to this “input vs. output” problem. One possible way to resolve this problem, at least partially, is to re-evaluate Δ*E* as an output of the FLEX calculation. However, this approach would suffer from various ambiguities regarding the Hubbard interaction strength and the definition of the renormalized Δ*E*. It is problematic since a quantitative comparison is required in between the theory and experiment, while only the qualitative comparison was made regarding *T*_*c*_ in Refs 26, 27, 28, 29.

In the present paper, we use a first-principles approach, exploiting the quasiparticle self-consistent *GW* (QSGW) method. It enables us to take into account the correlation effects beyond LDA/GGA. In this way, we can obtain a well-defined renormalized Δ*E* without introducing adjustable parameters.

In Ref. 17, a quantum chemical approach was adopted to evaluate the energy level offset between Cu-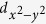 and 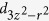 orbitals, where the correlation effects were taken into account within a cluster-based configuration-interaction-type calculation. A good agreement with the RIXS experiment was found by assuming the energy difference of ferromagnetically and antiferromagnetically ordered states to be 2*J*, where *J* is the antiferromagnetic coupling constant. Our approach is fairly different and is along the line of the first-principles band calculation as in Refs 26, 27, 28, 29. In the sense mentioned above, the calculated Δ*E* can be compared to the experiments, while it should not be regarded as an input parameter for the many-body calculation, because doing so would result in a partial double counting of the correlation effects. Still, the present approach can also provide a first-step hint toward obtaining a better “non-interacting” Hamiltonian that can be used as an input for the many-body calculation of superconductivity. In fact, it is known that the non-interacting Hamiltonian obtained from LDA has a problem when used as an input for the FLEX calculation, and the LDA/GGA estimation of Δ*E* for La_2_CuO_4_ is too small to account for the maximum *T*_*c*_ of 40 K in the La_2_CuO_4_^33^. In this context, it is worth pointing out that the *GW* method has been successfully applied to the many of strongly correlated materials in combination with, for example, dynamical mean field theory (DMFT)^34^,^35^,^36^,^37^.

## Results and Discussion

To our knowledge, there is no previous QSGW study for the cuprate band structure although it has been discussed conceptually^38^. Here we first examine the electronic structure and the two-band theory for the material-dependent T_*c*_ of a single layer cuprate. While the QSGW calculation produces notable differences in the band structure and Fermi surface from LDA, the two-band explanation for T_*c*_ still remains valid. QSGW results of model parameters are presented and compared to the RIXS data as well as the LDA calculations. It clearly shows that the parameters produced by QSGW are in better agreement with the experiment. Finally, we investigate the epitaxially strained La_2_CuO_4_ whose noticeable T_*c*_ increase has been previously reported. Two-band theory also works well for this situation.

### Electronic structure and the T_*c*_ of single layer compounds

**Figure 1:**
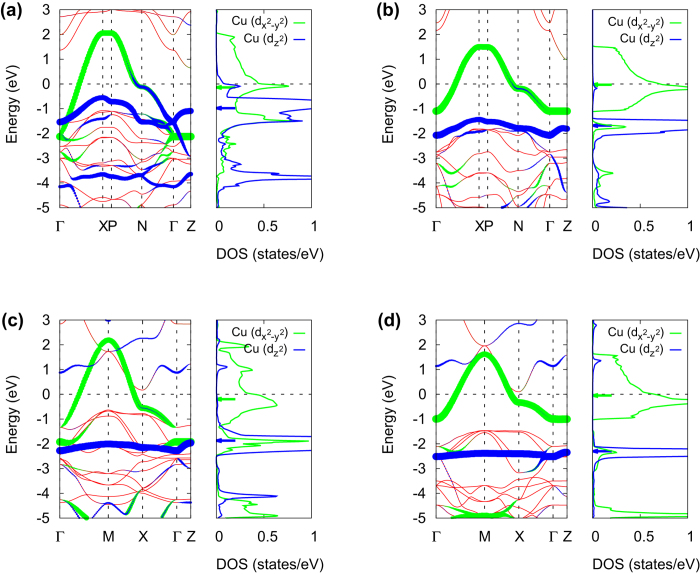
The band dispersion and PDOS of La_2_CuO_4_ (a,b) and HgBa_2_CuO_4_ (c,d) calculated by(a,c) LDA and (b,d) QSGW. The green and blue colors refer to the 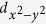 and 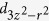 characters while the size of the colored dots represents their weight. The same color scheme was used for PDOS. Other bands than the two *e*_*g*_ states are represented by red color. The center of mass position of PDOS is marked by an arrow. Fermi energy is set to be 0.

Figure 1(a,b) show the band dispersion and projected density of states (PDOS) of La_2_CuO_4_ calculated by LDA and QSGW, respectively. The LDA result is in good agreement with the previous calculation (see, for example, Ref. 26 and 27). In the QSGW, several important differences are noted. First, the band width of both *e*_*g*_ orbitals are significantly reduced, by about 1.30 and 0.65 eV for 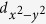 and 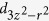, respectively, indicating that the band width overestimation (or effective mass underestimation) problem of LDA is somehow overcome by the QSGW procedure. Another key difference is that the separation between the 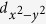 and 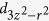 bands becomes larger in QSGW, as seen in Fig. 1(b). The 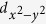 energy level, 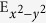, is shifted from −0.14 (LDA) to −0.03 (QSGW) while 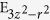 is from −0.98 (LDA) to −1.68 (QSGW) (indicated by the arrows in Fig. 1; see also Table 1). It is a factor of two difference in 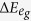; 0.84 eV in LDA and 1.66 in QSGW. The correct estimation of this quantity is important especially in the two-band theory for T_*c*_^26^,^27^,^28^,^29^. The large value of 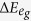 is indeed consistent with the RIXS data as will be discussed further below.

The same features are also found in HgBa_2_CuO_4_, as presented in Fig. 1(c,d). The 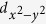 band width is reduced by ~0.75 eV in QSGW compared to LDA and its center position of PDOS moves slightly upward by 0.15 eV. While the 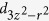 dispersion in this material is already quite small due to the thicker blocking layer, its band width in QSGW is further reduced. 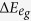 is 2.25 in QSGW, again noticeably larger than the LDA value of 1.67 eV (see Table 1).

In QSGW, the 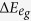 of both La_2_CuO_4_ and HgBa_2_CuO_4_ is enhanced compared to LDA/GGA. How does this affect the theoretical estimation of T_*c*_? First, it should be noted that these parameters cannot be directly adopted as the inputs for the FLEX calculation. This is because, in principle, the QSGW self energy should be partially subtracted before we put it into any of many-body calculations. While there is no well-defined prescription yet for this kind of ‘double-counting’ problem^39^, the “best” 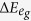 that should be adopted in the FLEX evaluation of T_*c*_ may be lying somewhere in between the QSGW and LDA/GGA values. This can provide better quantitative agreement with the experiment, especially in La_2_CuO_4_, for which the LDA/GGA value of 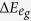 is found to be too small to account for *T*_*c*_ = 40 K.

**Figure 2:**
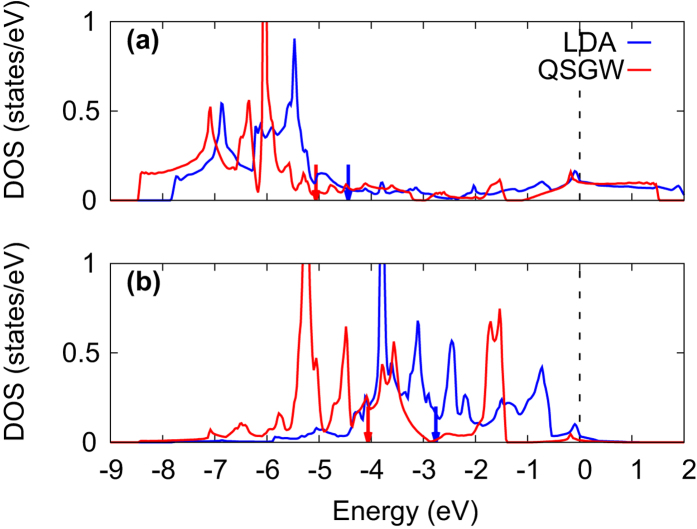
The calculated oxygen PDOS of La_2_CuO_4_ by LDA and QSGW. (**a**) In-plane oxygen 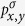 and (**b**) out-of-plane oxygen 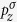 orbitals are plotted. The center of mass position of each PDOS is marked by an arrow. Fermi energy is set to be 0.

Oxygen states are also affected. Compared to LDA results, the O-2*p* levels obtained by QSGW are significantly lowered in energy, as indicated in Fig. 2. As summarized in Table 1, the center position of in-plane oxygen PDOS is located at −4.44 (−3.55) eV in LDA and at −5.06 (−4.05) eV in QSGW for La_2_CuO_4_ (HgBa_2_CuO_4_). The same feature is found for the apical O-*p*_*z*_ PDOS. As a result, the energy difference, ΔE_*p*_ = E_apical_ − E_inplane_, is changed from 1.68 (1.00) in LDA to 0.55 (−0.86) in QSGW for the case of La_2_CuO_4_ (HgBa_2_CuO_4_), see Table 1. The correct estimation of ΔE_*p*_ is also important for understanding T_*c*_ since it is an underlying quantity to determine 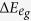 (≈ ΔE ≈ ΔE_*d*_ + ΔE_*p*_) in combination with other parameters. Note that our 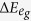 is different from ΔE_*d*_ and ΔE in Ref. 26, 27, 28, 29 where ΔE ≈ ΔE_*d*_ + ΔE_*p*_, 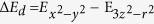, 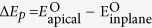, and all of the parameters are calculated from the maximally localized Wannier orbital analysis. The ΔE contains the contribution from oxygen hybridization. We note, however, that our 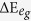 becomes effectively quite similar with ΔE in Ref. 26, 27, 28, 29 since we set our E_min,max_ to cover only the anti-bonding band complex. Actually one can make it almost equal, *i.e.,* 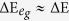, by fine-tuning the E_min,max_ range.

Some other changes produced by QSGW are also noted. The 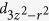 components in the bands below −1.5 eV in Fig. 1(a) are reduced in QSGW, and the free-electron-like bands at Γ and *Z* points above the Fermi energy are shifted upward. As the position of the *t*_2*g*_ complex is lowered (red color), the 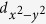 band has almost no mixture with other bands below the Fermi energy. Higher-lying La-4*f* bands (not shown) move further upward as has been previously noted in the nickelate systems^40^.

### Fermi surface

The shape of the Fermi surface is important for understanding cuprate superconductivity. For example, its nesting is crucial for the spin fluctuation pairing. Also, a notable correlation between the experimentally observed T_*c*_ at the optimal doping (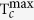) and the Fermi surface warping has been identified by Pavarini *et al.*^24^. Here we discuss the Fermi surface calculated by QSGW in comparison to the LDA result and experiment.

**Figure 3:**
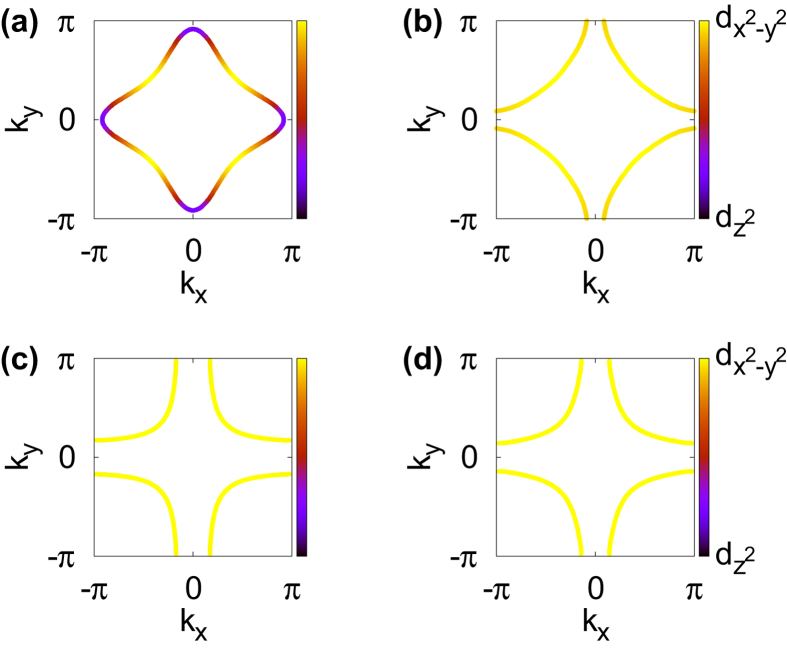
The orbital-resolved Fermi surfaces (*k*_*z*_ = 0 plane) at the reduced *e*_*g*_ band filling by 0.15 (hole doping): (**a**) La_2_CuO_4_ (LDA), (**b**) La_2_CuO_4_ (QSGW), (**c**) HgBa_2_CuO_4_ (LDA), and (**d**) HgBa_2_CuO_4_ (QSGW). The color represents the amount of 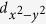 and 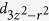 character.

The calculated Fermi surfaces are presented in Fig. 3; LDA ((a, c)) and QSGW ((b, d)). The hole doping is simulated by the rigid band shift method so that the electron occupation in *e*_*g*_ orbitals is reduced by 0.15*e* per unit cell. Notable features are found in the QSGW Fermi surface for La_2_CuO_4_. Contrary to the LDA result of Fig. 3(a), Fig. 3(b) has the pocket centered at (*π*, *π*) point as in HgBa_2_CuO_4_ Fermi surface (see Fig. 3(c,d)). This feature is in good agreement with ARPES data^41^ which also reports the pocket centered at (*π*, *π*) point. Further, the 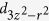-orbital character (dark purple) is significantly reduced and the 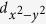 character (bright yellow) is dominant in the QSGW result, which is distinctive from the LDA in which the significant amount of 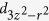 components are observed near (*π*, 0) and (0, *π*).

In the case of HgBa_2_CuO_4_, the difference between LDA and QSGW is less pronounced, see Fig. 3(c,d). While the QSGW Fermi surface is slightly more rounded, the overall shape is not much different. Since the 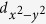 orbital character is dominant and 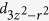 band is well separated from Fermi level already in LDA due to the thicker blocking layers enhancing two-dimensional feature, the LDA result is quite similar to QSGW.

### Comparison with RIXS

We now turn to the comparison with the RIXS data. Recently, Sala *et al.*^32^ successfully extracted the important model parameters for several different cuprate materials based on RIXS spectra. In this subsection, we examine the material dependent parameters by QSGW and compare them to the experimental values. With Ref. 32 as our main reference, we include two more compounds, namely, Sr_2_CuO_2_Cl_2_ and CaCuO_2_. Although Sr_2_CuO_2_Cl_2_ is also one of the single-layer cuprates, it has Cl—Cu—Cl bonding (instead of O—Cu—O) along *c*-axis and therefore the naive comparison of parameters such as 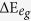 and ΔE_*p*_ in the line of T_*c*_ discussion may be misleading. This material is excluded in Ref. 26, 27, 28, 29 because of the same reason.

**Figure 4:**
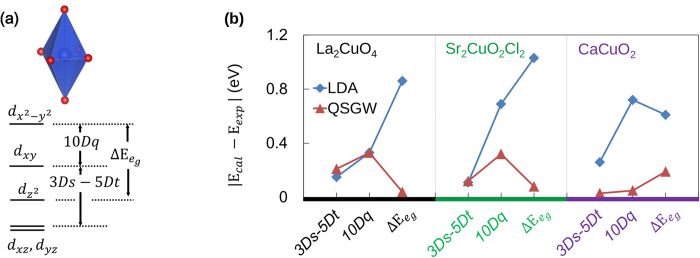
(**a**) The three model parameters for the comparison to Ref. 32. Note that 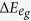 is denoted by 4*Ds* + 5*Dt* in Ref. 32. (**b**) The difference of the calculated model parameters (*E*_cal_) from the experiments (*E*_exp_).

**Figure 5:**
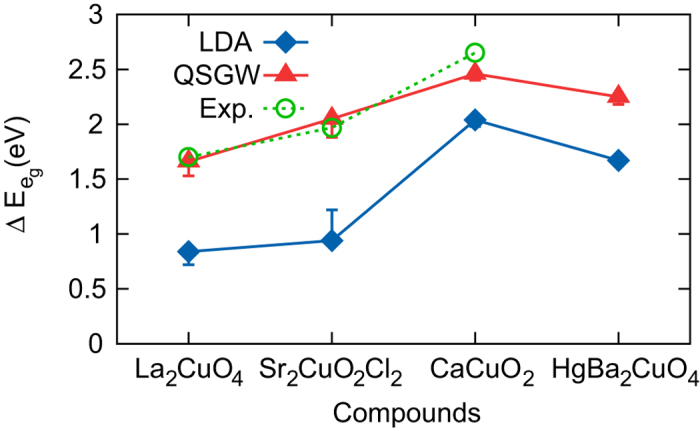
The values of 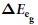 (or 4*Ds* + 5*Dt* in the notation of Ref. 32) estimated by LDA (blue squares), QSGW (red triangles), and RIXS data (green circles).

Our results are summarized in Table 1, Figs 4 and 5. The values of 10*Dq*, defined as the difference between two energy levels of 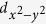 and *d*_*xy*_ (see Fig. 4(a)), are larger in the QSGW calculation by ~62–125% than the LDA values. While LDA underestimates 10*Dq* compared to the experiment, QSGW slightly overestimates, which is related to the tendency that Cu-*t*_2*g*_ bands are pushed down relative to *e*_*g*_, as was also observed in the previous QSGW calculations for other transition-metal oxides^40^,^42^,^43^. It is important to note that overall the QSGW result is in better agreement with experiment, as clearly seen in Fig. 4(b).

As noted in the above, according to the two-band theory by Sakakibara *et al.*^26^,^27^, the important parameter that governs T_*c*_ is 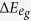 (or 4*Ds* + 5*Dt* in Ref. 32). Figures 4(b) and 5 clearly show that the calculated values of 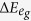 by QSGW are in excellent agreement with those from RIXS spectra; the difference is 2–8%. The LDA values are noticeably smaller than the experiments although the difference gets reduced in the higher T_*c*_ materials, CaCuO_2_ and HgBa_2_CuO_4_ (see Fig. 5). This can be taken as a strong support for the two-band theory in the sense that the LDA value of 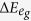 as an input for FLEX provides qualitative information of material dependence, while the 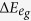 by QSGW already contains the correlation effect beyond LDA, being consistent with RIXS.

Another parameter deduced from RIXS in Ref. 32 is 3*Ds*—5*Dt*, the energy level difference between *d*_*xy*_ and *d*_*yz*,*zx*_. In this case, the LDA results are not much different from QSGW and experiment (see Fig. 4(b)).

### The effect of epitaxial strain

An interesting aspect found in the T_*c*_ trend of the cuprates is its significant enhancement in the thin film form. Locquet *et al.* reported^44^ that T_*c*_ can be controlled by epitaxial strain by about factor of two^45^. The underdoped La_2_CuO_4_ with its bulk T_*c*_ of 25 K exhibits a higher and lower T_*c*_ of ~49 K and 10 K when it is grown on SrLaAlO_4_ (SLAO) and SrTiO_3_ (STO) substrates, respectively^44^. It is therefore important to check whether the two-band theory is also consistent with this observation.

In order to simulate the tensile and compressive strain produced by STO and SLAO, we first optimized the *c* lattice parameter with two different in-plane lattice constants, *a*^STO^ = 3.905 and *a*^SLAO^ = 3.755 Å, for La_2_CuO_4_, which originally has *a*_0_ = 3.782 Å and *c*_0_ = 13.25 Å. As expected, the optimized out-of-plane parameters get smaller and larger under the tensile and compressive strain, respectively; 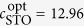 and 
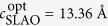
. As a result, the ratio between the out-of-plane and in-plane Cu—O distance, *r* = *d*_apical_/*d*_inplane_, is found to be 1.32, 1.28, and 1.24, for *a*^SLAO^, *a*_0_ and *a*^STO^, respectively.

**Figure 6:**
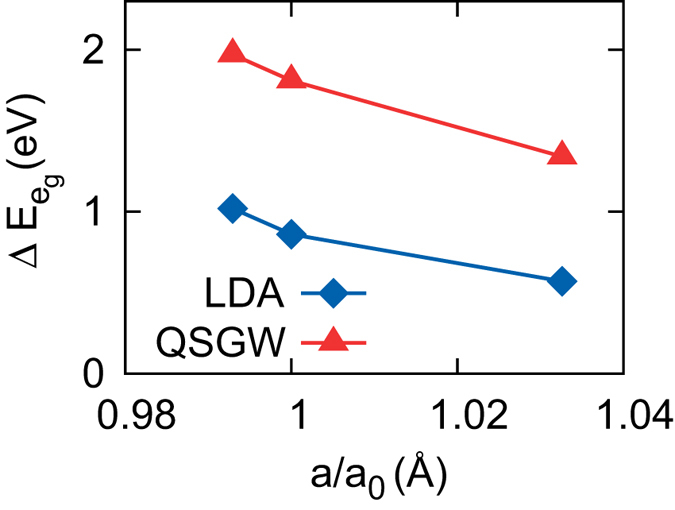
The calculated 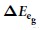 as a function of epitaxial strain. The *a*_0_ = 3.782 Å is the experimental value for bulk La_2_CuO_4_. The compressive and tensile strain are simulated with *a* = 3.755 and 3.905 Å considering the substrate of SrLaAlO_4_ and SrTiO_3_, respectively^44^.

The calculated values of 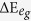 are plotted in Fig. 6. Both LDA and QSGW predict that 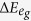 gets enhanced and reduced under compressive and tensile strain, respectively, which is consistent with the experimental observation^44^. The reduction of 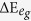 at a = *a*_STO_ is about 0.16 eV in both LDA and QSGW, and the enhancement at a = *a*_SLAO_ is 0.29 (LDA) and 0.47 eV (QSGW).

## Summary and Conclusion

Using the QSGW method, we re-examined the electronic structure of copper oxide high temperature superconducting materials. Several important features were found to have been captured by the *GW* procedure, such as effective mass enhancement. The shape and orbital character of the Fermi surface were also notably changed, especially for the case of La_2_CuO_4_, and they are in good agreement with the ARPES data^41^. Important model parameters including the key quantity for the two-band theory of T_*c*_, 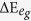, were examined, and the QSGW results were in excellent agreement with RIXS data.

The present study shows that the first-principles band calculation can quantitatively reproduce the experimental observation by taking into account the correlation effects beyond LDA. We emphasize that it is not inconsistent with the previous study by Sakakibara *et al.* which takes the LDA result as an input for the many-body calculation of superconductivity. While the QSGW result cannot be used as a direct input for the FLEX-type calculation because of the partial double-counting of the many-body correlation, the “best” non-interacting Hamiltonian, that can serve as an input, may lie somewhere in between the LDA and QSGW. Obtaining a well-defined non-interacting Hamiltonian is, therefore, an important future direction for the first-principles-based description of high-temperature superconductivity, and it may quantitatively resolve the problem of low T_*c*_ in La_2_CuO_4_ produced by the LDA input^33^.

## Methods

### Quasiparticle self-consistent *GW*

The QSGW^42^,^43^,^46^ calculates *H*_0_ (non-interacting Hamiltonian describing quasiparticles or band structures) and *W* (dynamically-screened Coulomb interactions between the quasiparticles within the random phase approximation) in a self-consistent manner. While the ‘one-shot’ *GW* is a perturbative calculation starting from a given *H*_0_ (usually from LDA/GGA), QSGW is a self-consistent perturbation method that can determine the one-body Hamiltonian within itself. The *GW* approximation gives the one-particle effective Hamiltonian whose energy dependence comes from the self-energy term Σ(*ω*) (here we omit index of space and spin for simplicity), and in QSGW, the static one-particle potential *V*^xc^ is generated as
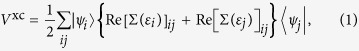
where *ε*_*i*_ and |*ψ*_*i*_〉 refer to the eigenvalues and eigenfunctions of *H*_0_, respectively, and Re[Σ(*ε*)] is the Hermitian part of the self-energy^42^,^43^,^46^. With this *V*^xc^, one can define a new static one-body Hamiltonian *H*_0_, and continue to apply *GW* approximation until converged. In principle, the final result of QSGW does not depend on the initial conditions. Previous QSGW studies, ranging from semiconductors^42^,^43^ to the various 3*d* transition metal oxides^42^,^43^,^47^ and 4*f*-electron systems^48^, have demonstrated its capability in the description of weakly and strongly correlated electron materials.

### Computation details

We used our new implementation of QSGW^49^ by adopting the ‘augmented plane wave (APW) + muffin-tin orbital (MTO)’, designated by ‘PMT’ 50,51, for the one-body solver. The accuracy of this full potential PMT method is proven to be satisfactory in the supercell calculations of homo-nuclear dimers from H_2_ through Kr_2_ with the significantly low APW energy cutoff of ~4 Ry, by including localized MTOs^51^. A key feature of this scheme for QSGW is that the expansion of *V*^xc^ can be made with MTOs, not APWs, which enables us to make the real space representation of *V*^xc^ at any *k* point.

We performed the calculations with the experimental crystal structures^52^,^53^,^54^,^55^, and used 10 × 10 × 10, 12 × 12 × 12, 12 × 12 × 8, and 14 × 14 × 14 *k* points for LDA calculations of Sr_2_CuO_2_Cl_2_, La_2_CuO_4_, HgBa_2_CuO_4_, and CaCuO_2_, respectively. As for QSGW calculations, in order to reduce the computation cost, the number of *k* points were reduced to be 5 × 5 × 5, 7 × 7 × 7, 8 × 8 × 4, and 8 × 8 × 8 for the first Brillouin zone of Sr_2_CuO_2_Cl_2_, La_2_CuO_4_, HgBa_2_CuO_4_, and CaCuO_2_, respectively. The MTO radii used in our calculations were as follows: (i) 1.58, 1.04, 0.89, and 1.38 Å for Sr, Cu, O, and Cl in Sr_2_CuO_2_Cl_2_, (ii) 1.43, 0.97, and 0.86 Å for La, Cu, and O in La_2_CuO_4_, (iii) 1.10, 1.59, 1.05, and 0.83 Å for Hg, Ba, Cu, and O in HgBa_2_CuO_4_, and (iv) 1.54, 1.01, and 0.86 for Ca, Cu, and O in CaCuO_2_.

Many of the key parameters in this study are defined in terms of the energy levels of each orbital, such as 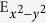 and 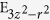. To quantify them we simply take the center of mass position of PDOS:
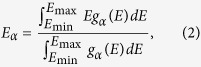
where *g*_*α*_(*E*) is PDOS for a given orbital *α*. An ambiguity is inevitably introduced in determining E_min,max_, and we set the range to cover the whole antibonding band complex for Cu-*e*_*g*_ states. (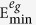, 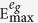) for La_2_CuO_4_ is (−1.95 eV, 2.05 eV) in LDA and (−2.20, 1.55) in QSGW. For HgBa_2_CuO_4_, the band dispersion changes and the values of 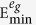 and 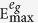 are redefined accordingly: (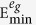, 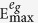) = (−2.40, 2.50) in LDA and (−2.55, 1.65) in QSGW. If we choose two different ranges for two *e*_*g*_ orbitals to include only the main peak of each orbital PDOS, we can actually produce the better agreement with the numbers in the previous study by Sakakibara *et al.* where the levels are defined using maximally localized Wannier function method^26^,^27^. Even if the ranges are set to cover the whole window of Cu-*e*_*g*_ bands including bonding parts, the trend reported in this work does not change. The same is true for O-2*p* and Cu-*t*_2*g*_ levels. In other words, none of the reasonably defined energy ranges change our conclusion, and the values are well compared with those reported in the previous study using a maximally localized Wannier function^26^,^27^.

## Additional Information

**How to cite this article**: Jang, S. W. *et al.* Quasiparticle self-consistent *GW* study of cuprates: electronic structure, model parameters, and the two-band theory for T_c_. *Sci. Rep.* **5**, 12050; doi: 10.1038/srep12050 (2015).

## Figures and Tables

**Table 1:** The calculated parameters by LDA and QSGW.

	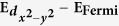 **(eV)**		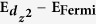 **(eV)**		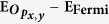 **(eV)**		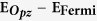 **(eV)**		
	**LDA**	**QSGW**	**LDA**	**QSGW**	**LDA**	**QSGW**	**LDA**	**QSGW**	
Sr_2_CuO_2_Cl_2_	−0.29	0.01	−1.23	−2.05	−4.67	−4.92	None	None	
La_2_CuO_4_	−0.14	−0.03	−0.98	−1.68	−4.44	−5.06	−2.76	−4.06	
HgBa_2_CuO_4_	−0.20	−0.05	−1.87	−2.30	−3.55	−4.05	−3.00	−4.91	
CaCuO_2_	−0.30	−0.17	−2.34	−2.63	−3.67	−4.05	None	None	

	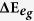 **(4Ds + 5Dt) (eV)**			**3Ds** − **5Dt (eV)**			**10Dq (eV)**		
	**LDA**	**QSGW**	**Exp (**32)	**LDA**	**QSGW**	**Exp (**32)	**LDA**	**QSGW**	**Exp (**32)
Sr_2_CuO_2_Cl_2_	0.94	2.05	1.97	0.22	0.21	0.33	0.81	1.82	1.50
La_2_CuO_4_	0.84	1.66	1.70	0.17	0.11	0.32	1.47	2.13	1.80
HgBa_2_CuO_4_	1.67	2.25	None	0.42	0.42	None	0.90	1.57	None
CaCuO_2_	2.04	2.46	2.65	0.05	0.28	0.31	0.92	1.69	1.64

The *p*_*x,y*_ is from the in-plane oxygen, and p_*z*_ from the out-of-plane. The definitions of parameters can be found in Fig. 4. The experimental values are taken from Ref. 32.
